# Neurogenic potential of dental pulp stem cells isolated from murine incisors

**DOI:** 10.1186/scrt419

**Published:** 2014-02-27

**Authors:** Kylie M Ellis, David C O’Carroll, Martin D Lewis, Grigori Y Rychkov, Simon A Koblar

**Affiliations:** 1 Adelaide Centre for Neuroscience Research, University of Adelaide, Adelaide, South Australia, Australia; 2 School of Medical Sciences, University of Adelaide, Adelaide, South Australia, Australia; 3 School of Molecular and Biomedical Science, University of Adelaide, Adelaide, South Australia, Australia; 4 School of Medicine, University of Adelaide, Adelaide, South Australia, 5005, Australia; 5 Stroke Research Programme, University of Adelaide, Adelaide, South Australia, Australia

## Abstract

**Introduction:**

Interest in the use of dental pulp stem cells (DPSC) to enhance neurological recovery following stroke and traumatic injury is increasing following successful pre-clinical studies. A murine model of autologous neural stem cell transplantation would be useful for further pre-clinical investigation of the underlying mechanisms. However, while human-derived DPSC have been well characterised, the neurogenic potential of murine DPSC (mDPSC) has been largely neglected. In this study we demonstrate neuronal differentiation of DPSC from murine incisors *in vitro*.

**Methods:**

mDPSC were cultured under neuroinductive conditions and assessed for neuronal and glial markers and electrophysiological functional maturation.

**Results:**

mDPSC developed a neuronal morphology and high expression of neural markers nestin, ßIII-tubulin and GFAP. Neurofilament M and S100 were found in lower abundance. Differentiated cells also expressed protein markers for cholinergic, GABAergic and glutaminergic neurons, indicating a mixture of central and peripheral nervous system cell types. Intracellular electrophysiological analysis revealed the presence of voltage-gated L-type Ca^2+^ channels in a majority of cells with neuronal morphology. No voltage-gated Na^+^ or K^+^ currents were found and the cultures did not support spontaneous action potentials. Neuronal-like networks expressed the gap junction protein, connexin 43 but this was not associated with dye coupling between adjacent cells after injection of the low-molecular weight tracers Lucifer yellow or Neurobiotin. This indicated that the connexin proteins were not forming traditional gap junction channels.

**Conclusions:**

The data presented support the differentiation of mDPSC into immature neuronal-like networks.

## Introduction

Since their discovery as a source of multipotent adult human stem cells by Gronthos *et al.*[1], numerous groups have confirmed the potential of dental pulp stem cells (DPSC) to differentiate into multiple neural crest-lineage cell types [2-4]. Previous studies in our laboratory and others have demonstrated the neural potential of human-derived DPSC *in vitro*[2,5] and in *vivo*[6-8]. Human DPSC were found to express neural markers following injection into the rat and embryonic chick brain [7,8] and also induced endogenous responses through paracrine effects [6,9,10]. In the chick embryo, human DPSC induced neuroplasticity of the highly structured trigeminal ganglion [6] and promoted the recruitment, proliferation and neural differentiation of endogenous precursors in the mouse brain [9]. Interestingly, pre-differentiation of human DPSC promoted greater cell survival and neural differentiation following rat cortical lesion [7], which could be reflected therapeutically with greater functional recovery.

Given their potential for autologous transplantation and therapeutic applications in dental engineering and neurological disease treatment, the focus to date has been on applications for human-derived DPSC. The cellular and molecular mechanisms underlying recovery in pre-clinical studies of varied animal models of disease are poorly understood. Xenotransplantation is often problematic (that is, human DPSC injected into rodents) due to immune rejection. The mouse is a fundamentally important animal model in relation to understanding human disease, pre-clinical testing, and transgenic potential to gain better knowledge of mechanisms of action. A murine model of autologous DPSC transplantation would, therefore, be of great utility.

Like their human counterparts, rodent DPSC show neural crest multipotentiality [11-14]. However, a distinction has emerged between DPSC from murine molar and incisor teeth. While they both possess osteo-dentin and adipocyte differentiation potential, erupted murine molars, but not incisors, have been found to have chondrocytic potential [11-13,15]. Janebodin *et al*. [13] have described the expression of neuronal, oligodendrocyte and glial markers after *in vitro* differentiation of murine molar DPSC. To the best of our knowledge neural differentiation of incisor murine DPSC (mDPSC) has not yet been attempted and could offer an easily accessible source of DPSC for pre-clinical studies. Work by two other groups suggests that rodent incisor DPSC do have neurogenic potential through the successful formation of cells with neuronal-like multipolar morphology that expressed neuronal markers *in vitro*[16,17] and the promotion of nerve regeneration *in vivo* using rat incisor DPSC [18]. Neither study reported electrophysiological properties of the rat DPSC after neuronal differentiation.

Herein, we report the *in vitro* neuronal development of DPSC isolated from murine incisors using a neural differentiation methodology found to generate functional neurons from human DPSC [5]. We found species-specific differences between human and mouse cells and demonstrated that mDPSC develop characteristics suggesting their differentiation into immature neural-like cells. Unique to our study is the interrogation of the neuronal characteristics of mDPSC-derived cells using electrophysiological methodologies, which is fundamental to understanding neuronal function.

## Methods

### mDPSC isolation and culture

Incisors from adult BalbC mice were removed and their pulp exposed to enzymatic digestion with 3 mg/mL collagenase type I and 4 mg/mL dispase in PBS for one to two hours at 37°C with 5% CO_2_. The resulting solution was centrifuged at 200 × g for five minutes, the supernatant and enzymes removed and the remaining cells cultured in mesenchymal stem cell medium [19] containing alpha-modified Eagle’s medium (α-MEM) supplemented with 10% foetal bovine serum (FBS, Invitrogen, Mulgrave, Victoria, Australia), 1x GlutaMAX (Gibco, Mulgrave, Victoria, Australia), 100 μM L-ascorbate-2-phosphate (Wako, Neuss, Germany), 50 U/mL penicillin and 50 μg/mL streptomycin (Invitrogen), and dental pulp stem cells were allowed to adhere to the plastic base. Floating debris could subsequently be removed.

### Ethics statement

Animal ethics was approved by the University of Adelaide Animal Ethics Committee (S-2009-159).

### mDPSC neuronal differentiation

**Figure 1:**
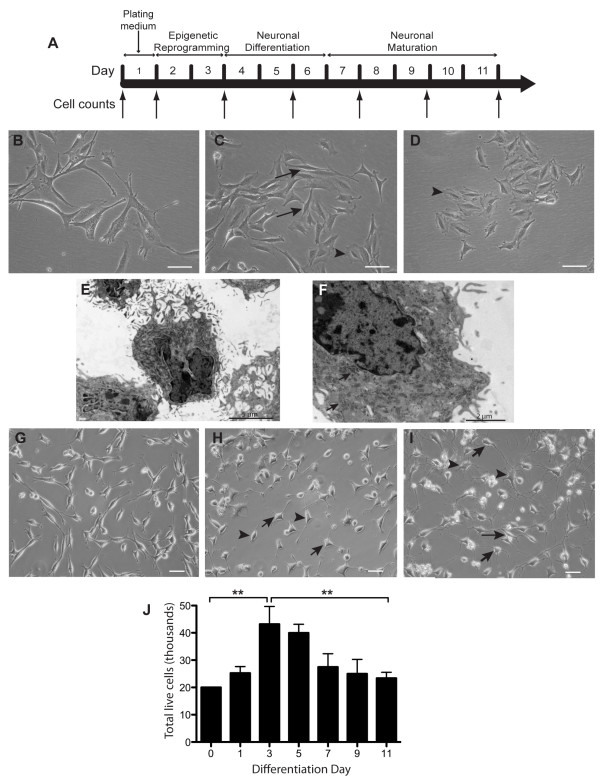
**Timeline, phenotype and survival of differentiating mDPSC. A)** Timeline of neuronal induction protocol with successive medium changes through plating, epigenetic reprogramming, neuronal differentiation and neuronal maturation phases. Cell counts were performed at days 0, 1, 3, 5, 7, 9 and 11 to determine cell viability. **B-D)** Undifferentiated mDPSC had a fibroblast-like morphology *in vitro*. Some displayed longer processes (**B**,**C**, arrows) while a smaller population had a webbed soma (**C**,**D** arrowheads). **E-F)** Representative electron micrographs of undifferentiated mDPSC showing a complex ultrastructure with irregularly-shaped nuclei, widespread endoplasmic reticula (arrows) and microvilli-like projections. **G-I)** Representative bright field images of mDPSC at days 1 **(G)**, 5 **(H)** and 11 **(I)** of neuronal differentiation. Cells began to develop short, thin processes by day 5 with bipolar (arrowheads) and multiprocessor (short arrows) morphologies becoming evident. By day 11 mDPSC-derived neural cells had three distinct morphologies: bipolar (long arrow), multiprocessor neural-like cells (arrows) and large, flat multiprocessor glial-like cells (arrowheads). **J)** Average number of live cells throughout differentiation (n = 3 differentiation batches). Cells showed a high rate of proliferation until day 3 and steadily reduced until significantly fewer cells were alive at day 11. An average of 23,000 from the original 20,000 plated per well were alive at day 11. Scale bars = 50 μm. **P <0.01.

mDPSC were seeded at 20,000 cells/cm^2^ onto laminin (0.02 mg/mL, Gibco) and poly-L-lysine (0.01%) coated glass coverslips and were induced to differentiate based on a protocol previously described [5] (Figure 1A). Cells were first maintained in plating medium containing 1:1 (D)MEM/F-12 (Gibco) supplemented with 2.5% FBS, 50 U/mL penicillin and 50 μg/mL streptomycin for 24 hours. They then underwent epigenetic reprogramming for 48 hours with the addition of 10 μM 5-azacytidine, 1 mM dbcAMP and 10 ng/mL mouse-specific fibroblast growth factor-2 (FGF-2, ProSpec, Niss-Ziona, Israel) to the basic plating medium. Cells were then washed with PBS and induced with a neural differentiation medium containing 250 μM 3-isobutyl-1-methylxanthine (IBMX), 50 μM forskolin, 1% insulin-transferrin-selenium (ITS), 30 nM phorbol 12-myristate 13-acetate (TPA), 30 ng/mL neurotrophin-3 (NT-3, ProSpec), 10 ng/mL mouse-specific nerve growth factor (NGF), 10 ng/mL FGF-2 in 1:1 (D)MEM/F12 for three days. Finally, cells were rinsed again with PBS before the addition of a neuronal maturation medium for three to seven days which consisted of 1% N2 and B27 supplements (Gibco), 30 ng/mL NT-3, 1 mM dbcAMP in 1:1 (D)MEM/F12. Cell counts were performed by trypan blue exclusion at days 0, 1, 3, 5, 7, 9 and 11 (Figure 1A). Three technical replicates were assessed per time point for three differentiation batches. Statistical analysis of cell proliferation and attrition was performed with one-way analysis of variance with Bonferroni post hoc analysis. Unless otherwise stated, reagents were sourced from Sigma-Aldrich, Sydney, New South Wales, Australia.

### Immunohistochemistry

mDPSC cultures were fixed either undifferentiated or at day 11 of neuronal differentiation with 4% formaldehyde for 20 minutes. Cells were rinsed then permeabilised with 3% H_2_O_2_, 10% methanol in PBS for ten minutes and subsequently washed three times with PBS. Due to high background staining of pilot cultures, mDPSC were blocked at 4°C overnight with 1% bovine serum albumin, 3% horse serum and 3% donkey serum in 0.3% Triton X-100 in PBS (PBS-Tx). Cultures were then incubated with primary antibody diluted in block solution overnight at 4°C. Cells were again rinsed three times with 0.3% PBS-Tx and incubated with secondary antibody for one hour at room temperature with gentle shaking. After rinsing, cultures underwent counterstaining or coverslips were removed from wells and mounted onto slides with ProLong Gold with 4',6-diamidino-2-phenylindole (DAPI) (Invitrogen). Images were taken with a Leica SP5 scanning confocal microscope and percentage expression of each marker was determined by manual counts of four representative fields of view. Data were assessed using two-way analysis of variance (ANOVA) with Bonferroni *post hoc* test.

Primary antibodies raised in mouse targeted ßIII tubulin (Millipore, Kilsyth, Victoria, Australia), neurofilament – medium chain (NF-M, Zymed, Mulgrave, Victoria, Australia) and S100 (Chemicon, Millipore, Kilsyth, Victoria, Australia). Antibodies raised in rabbit targeted nestin (Abcam, Waterloo, New South Wales, Australia), glial fibrillary acidic protein (GFAP; 1:1000, DAKO, Noble Park, Victoria, Australia), connexin43 (Invitrogen), glutamic acid decarboxylase 65/67 (GAD65/67; Millipore), choline acetyltransferase (ChAT; Biosensis, Thebarton, South Australia, Australia), tyrosine hydroxylase (Chemicon) and vesicular glutamate transporter 2 (vGlut2; Synaptic Systems, Goettingen, Germany). Secondary antibodies used were Alexa555 anti-mouse (Invitrogen), donkey Cy3 anti-rabbit (Jackson, West Grove, PA, USA) and rabbit Cy3-labelled streptavidin (Invitrogen). Non-specific fluorescence was determined by applying each secondary antibody alone, omitting the primary antibody and used as a control. Primary and secondary antibodies were used at 1:500 dilution unless otherwise stated.

### Intracellular electrophysiology

#### ***Patch clamp analysis***

Whole-cell voltage clamp analysis of mDPSC was performed at room temperature using a computer-based amplifier (EPC-9, HEKA Electronics, Lambretch/Pfalz, Germany) and PULSE software (HEKA Elektronik, Lambrecht/Pfalz, Germany). Patch pipettes were pulled from borosilicate glass and fire-polished with resistance ranging from 3 to 6 MΩ. Internal pipette solution contained 135 mM Cs-glutamine, 5 mM CaCl_2_, 5 mM MgCl_2_, 10 mM HEPES, 200 μM GTP, 5 mM ATP, 10 mM EGTA and pH adjusted to 7.3 with NaOH. The calculated internal free Ca^2+^ concentration was approximately 100 nM. A standard bath solution with 10 mM CaCl_2_, 140 mM NaCl, 4 mM Cs-glutamine, 2 mM MgCl_2_ and 10 mM HEPES, adjusted to pH 7.4 was used. Differentiated mDPSC with a neuronal morphology that could be classified as either isolated from other cells or clustered (see Results) were targeted. Holding potential was set at -60 mV and 500 ms voltage steps ranging between -40 and 50 mV were applied in 10 mV increments to record membrane currents. Cells were interrogated for evidence of voltage-gated Na^+^, Ca^2+^ and K^+^ currents as well as sensitivity to 100 nM tetrodotoxin (TTX) and 10 mM Ba^2+^ to block or enhance currents through Na^+^ and Ca^2+^ channels, respectively. To record K^+^ currents, Cs-glutamine in the internal solution and CsCl in the bath solution were replaced with K-Glutamine and KCl, respectively. Furthermore, L-type Ca^2+^ currents were recorded from undifferentiated and differentiated mDPSC in response to 100 ms voltage ramps from -120 to +120 mV to compare the current amplitude between cell types. Pharmacological agents were introduced through a gravity-fed perfusion system. The capacitance of each cell was measured using the automatic capacitance compensation routine of EPC-9 amplifier. Series resistance did not exceed 20 MΩ and was not compensated for.

#### ***Neurobiotin and Lucifer yellow injection***

Two percent neurobiotin and 10 μg/mL Lucifer yellow were injected into clustered mDPSC with neuronal morphology by a whole cell patch clamp technique. A patch was maintained with the target cell for five minutes to allow the internal pipette solution to diffuse into the patched and surrounding connected cells. Cells were immediately imaged with an epifluorescent microscope to visualise Lucifer yellow and then fixed with 4% paraformaldehyde (PFA) for 20 minutes prior to immunohistochemical staining for neurobiotin. Cultures were washed with PBS, permeabilised with 0.3% PBS-Tx, then counterstained with Cy3-labeled streptavidin. Cells were imaged on a Zeiss AxioImager Z1 ApoTome microscope.

#### ***Transmission electron microscopy (TEM)***

Undifferentiated mDPSC (3 × 10^6^) were liberated with trypsin from two T75 flasks and centrifuged at 200 × g for two minutes. Cells were resuspended in 1.5 mL 1.25% Gluteraldehyde (ProSciTech; Kirwan, Queensland, Australia) and 4% PFA (Sigma Aldrich; Sydney, New South Wales, Australia) (EM fixative) and stored at 4°C for 24 hour. All subsequent preparation was performed in a fume hood. Cells were washed with 4% sucrose in PBS for five minutes, then post fixed in 2% Osmium tetroxide (ProSciTech; Kirwan, Queensland, Australia) for 45 minutes. Cells were next dehydrated by serial incubations with 70%, 90%, 95% and 100% ethanol, then incubated in Propylene oxide (ProSciTech; Kirwan, Queensland, Australia) for 20 minutes. A 1:1 mixture of propylene oxide:resin was applied for one hour then 100% resin, 2× one hour. Cells were then embedded in fresh 100% resin and polymerized at 70°C for 24 hours. The resin mixture was made of 10 mL Procure (ProSciTech; Kirwan, Queensland, Australia) 812, 6 mL Araldite (ProSciTech; Kirwan, Queensland, Australia) 502, 22 mL DDSA (ProSciTech; Kirwan, Queensland, Australia) and 560 μL DMP (ProSciTech; Kirwan, Queensland, Australia). Ultrathin sections were taken and imaged on a Philips CM100 TEM.

#### ***Microelectrode arrays***

Microelectrode arrays (MEAs) and recording stage were supplied by MultiChannel Systems (MCS, Reutlingen, Germany). Each electrode array contained 59 active titanium nitride electrodes arranged in an 8 × 8 grid with the corner electrodes absent and one electrode used as an electrical reference. Electrode diameter was 30 μm with an inter-electrode distance of 200 μm. Signal acquisitions were managed under MC_DataTool Software software control and sampled at a frequency of 50 kHz.

#### ***Microelectrode array electrophysiology***

mDPSC were seeded onto the centre of microelectrode arrays (n = 12 cultures) in a 40 μL droplet containing 20,000 cells and were kept in a humidified incubator at 37°C with 5% CO_2_. MEAs were sealed with a Teflon membrane lid (MCS) to minimize evaporation. The cells were allowed to settle for one hour then flooded with 1 mL culture medium. Cultures underwent neuronal induction according to the protocol described above. The external electrophysiology of the cultures was assessed from differentiation day 10 to determine spontaneous array-wide activity. To record from cultures, MEAs were placed in an electrically grounded recording stage and allowed to settle for 30 minutes. Three 100 second recordings were taken from all 59 electrodes for each MEA. TTX (10 μM) was added to a subset of cultures to inhibit any action potentials (n = 4 cultures). As a positive control, neuronally differentiated murine embryonic stem cells (46C, n = 2 cultures) and cortical neurons (n = 2) were also seeded onto MEAs and assessed for spiking activity (see Additional file 1). To determine tonic activity of the system, recordings were made with PBS only on MEAs (n = 4). Data were analysed with Spike 2 software.

#### ***MEA data analysis***

MEA data were subsequently analysed using Spike2 and Matlab software. A low pass filter of 4,000 Hz was applied to all MEA traces prior to analysis. The standard deviation of noise was then calculated for each individual electrode trace and a spike detection threshold set at five times this standard deviation. Only supra-threshold events with a physiologically relevant shape and duration were detected as events according to Spike2 settings and each event detected was closely scrutinized for reliability. The spike rate, amplitude and duration of differentiated mDPSC events were compared to controls and assessed statistically by one-way ANOVA with Tukey’s *post hoc* analysis. Only electrodes with three or more events per 100 second interval were considered active for analysis purposes.

## Results

### Undifferentiated DPSC from murine incisors

Cultured mDPSC displayed a heterogeneous phenotype *in vitro.* The cells were adherent and the majority had large nuclei with fibroblast-like soma and projections (Figure 1B and C). A smaller proportion of cells displayed a webbed-like soma (Figure 1D). The proliferation of mDPSC appeared rapid with the population doubling in three days. Transmission electron microscopy demonstrated an elaborate ultrastructure of mDPSC with large irregularly shaped nuclei (Figure 1E), extensive rough endoplasmic reticula (Figure 1F, arrows) and an intricate outer membrane of microvilli-like projections.

### Characterisation of mDPSC following neural induction

mDPSC robustly and reproducibly differentiated into neural phenotypes using the described neuronal induction protocol (Figure 1A). During the epigenetic reprogramming (ER) stage mDPSC developed rounded phase-bright soma with two distinct morphologies: those cells with bipolar processes and cells with multiple processes (Figure 1G and H, arrows). We found that during the plating and ER stages there was a significant increase in cell number from an average at plating of 20,000 cells to 43,000 by the third day of the neuronal induction protocol, likely due to continued cell proliferation (Figure 1J). Within 24 hours of changing to the neuronal differentiation (ND) stage, which includes specific growth factors (FGF, NT-3 and NGF), cAMP and PKC agonists as well as the removal of serum, the cells had differentiated into more neuronal-like morphologies (Figure 1H). During the ND and neuronal maturation (NM) stages there was marked cell death with numbers stabilising to approximately 23,000 cells by the end of NM. There was a significant change in cellular morphology during NM into three distinct types: rounded and phase-bright soma with multiple long processes (Figure 1I, short arrows), smaller phase-bright bipolar cells (long arrow), and large non-phase-bright flatter cells with more diffuse multi-processes (arrowheads). The former two cell types described represented neuronal-like morphologies. Overall, the cells matured into a complex neuritic network with approximately two thirds having a neuronal-like morphology.

**Figure 2:**
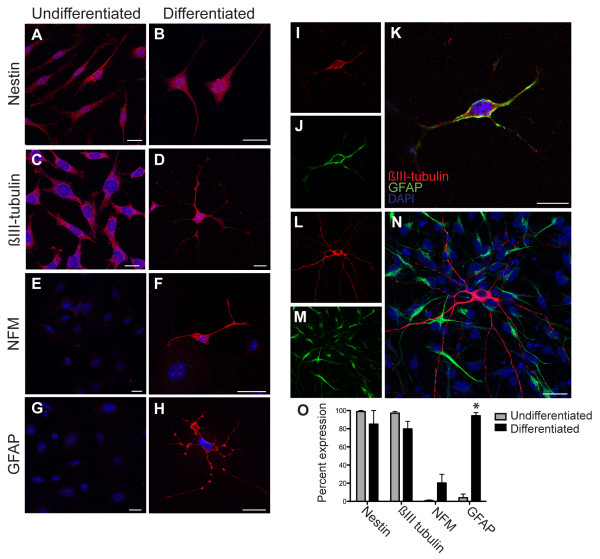
**Differentiated mDPSC express neuronal and glial markers. A-H)** Representative immunohistochemistry images of undifferentiated mDPSC showing strong expression of nestin and ßIII tubulin but low levels of NFM and GFAP. At day 11 of neuronal differentiation mDPSC retained strong nestin and ßIII tubulin expression **(A-D)**. NFM and GFAP expression was markedly increased following neural induction **(E-H)**. **I-K)** The majority of cells co-expressed ßIII tubulin and GFAP. **L-N)** Validation of co-expression result using the same ßIII tubulin and GFAP antibodies applied to murine cortical cells, which demonstrates the expected distinction between neurons and glia. **O)** Quantification of neural IHC. The relative expression of each neural marker before and after differentiation, counted as percent expression per four representative fields of view at 20x magnification. Scale bar = 25 μm. *P <0.05. GFAP, glial fibrillary acidic protein; IHC, immunohistochemistry; mDPSC, murine dental pulp stem cells; NFM, neurofilament-medium chain.

At day 11 of induction, mDPSC expressed a variety of neuronal and glial proteins. Immunohistochemistry demonstrated the majority of differentiated cells expressed nestin and ßIII tubulin (85% and 79%, respectively; Figure 2B and D). In regard to nestin we found no significant change in expression in comparison to undifferentiated mDPSC with almost all cells expressing this neural precursor protein (Figure 2A, B and O). ßIII tubulin expression reduced from 97% in undifferentiated mDPSC (Figure 2C) to 79% following induction (Figure 2D and O). There was a marked co-expression of nestin and ßIII tubulin in undifferentiated and differentiated mDPSC. Using a mature neuronal marker, neurofilament-medium chain (NFM) [20], expression increased considerably from 2% in the undifferentiated mDPSC up to 20% following induction (Figure 2E and F). GFAP expression was seen in 4% of undifferentiated mDPSC and increased significantly to 94% in differentiated cells (Figure 2G, H and O). Unexpectedly, the majority of differentiated cells that expressed ßIII-tubulin also co-expressed GFAP (Figure 2I-K). To validate this finding of co-expression the same antibodies against ßIII-tubulin and GFAP were used in cultures of primary E19 murine cortical cells and clearly indicated discrete expression of these proteins with respect to their neural cell types (Figure 2L-N).

**Figure 3:**
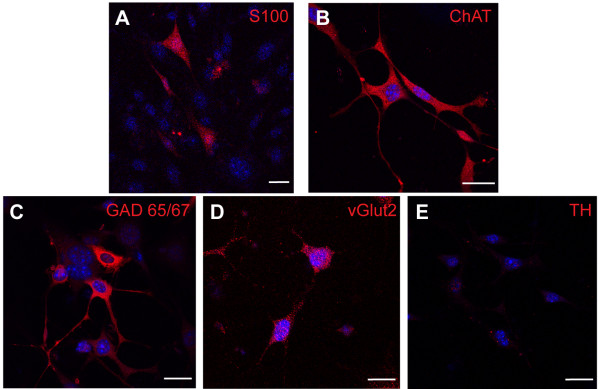
**Differentiated mDPSC produce central and peripheral nervous system markers.** At day 11 of differentiation mDPSC express central and peripheral nervous system glial marker, S100 **(A)**, and acetylcholine-specific enzyme, ChAT **(B)**. mDPSC also show positive expression of GAD 65/67 **(C)** and vGlut2 **(D)** but not TH **(E)**. Scale bar = 25 μm. ChAT, choline acetyltransferase; GAD65/57, glutamic acid decarboxylase 65/67; mDPSC, murine dental pulp stem cells; TH, tyrosine hydroxylase; vGlut2, vesicular glutamate transporter 2.

We next investigated neurally differentiated mDPSC for a range of antigens expressed by mature neural cells to determine whether the expression patterns were consistent with peripheral or central nervous system cell types. We found that 5% of mDPSC at induction day 11 expressed the central and peripheral glial marker, S100, and 87% were positive for acetylcholine specific neurons, ChAT (Figure 3A and B, respectively). There was also specific expression of markers for GABAergic (GAD65/67, 5%) and glutamatergic neurons (vGlut2, 15%), but not dopaminergic neurons with a lack of tyrosine hydroxylase immunoreactivity (Figure 3C, D and E, respectively).

### Neural network properties of differentiated mDPSC

**Figure 4:**
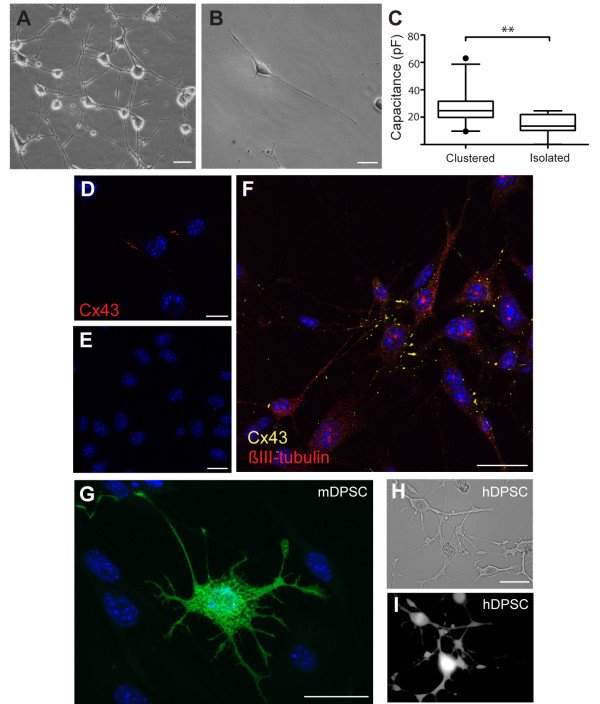
**Network connectivity of differentiated mDPSC.** Representative images of differentiated mDPSC in a cluster of many cells **(A)** and as an isolated unit **(B)**. **C)** Capacitance of clustered cells (n = 23) was significantly greater than isolated cells (n = 12) measured by whole cell patch clamp analysis. IHC shows mDPSC expression of connexin 43 (Cx43) in differentiated **(D)** but not undifferentiated cells **(E)**. **F)** Cx43 (yellow) is most highly expressed within clusters of ßIII-tubulin (red) positive differentiated mDPSC (iii). **G)** Image of a clustered mDPSC injected with Lucifer yellow and neurobiotin does not show any dye coupling with adjacent cells. Lucifer yellow injection into a clustered differentiated human DPSC does show dye coupling through numerous adjacent cells observed under bright field **(H)** and ultraviolet light **(I)**. Scale bar = 25 μm. **P <0.01. IHC, immunohistochemistry; mDPSC, murine dental pulp stem cells.

Differentiated mDPSC demonstrated variable properties indicative of neural networks. Cells were subject to whole cell patch clamp analysis and the results were grouped into neuronal-like cells that were either clustered (Figure 4A) or isolated from each other (Figure 4B). Amplifier-reported capacitance of clustered cells (M = 26.64 ± 12, n = 23 cells) was significantly greater than that of isolated cells (M = 14.32 ± 7.2, n = 12; *P* <0.01, Figure 4C). However, the reported capacitance of clustered cells was likely a gross underestimation of actual values due to the amplifier limitations in compensating for such large current dissipation. Nevertheless, the reported capacitance of isolated cells was accurate and demonstrated the distinction between the classes of differentiated mDPSC.

The nature of cell-cell contacts within clusters was investigated further using immunohistochemistry. Connexin 43 (Cx43), a common gap junction protein, was expressed widely in differentiated (Figure 4D) but not undifferentiated mDPSC (Figure 4E). Cx43 was more abundantly expressed in cell clusters, as would be expected (Figure 4F). We did not find synapsin 1 expression in differentiated cells (data not shown).

To assess the functionality of these gap junctions, Lucifer yellow and neurobiotin tracer dye were injected into a single cell through a patch pipette and allowed to spread through membrane pores or gap junctions of sufficient size (n = 9 patched cells). Surprisingly, there was no evidence of Lucifer yellow or neurobiotin spread to adjacent cells in differentiated mDPSC cultures indicating that gap junctions were not permeable to small molecules (Figure 4G). In contrast, extensive Lucifer yellow dye spread was seen following injection into a single human DPSC after 14 days of the same neuronal differentiation protocol (n = 3 human DPSCs injected, Figure 4H and I). These data indicate a physiological distinction between species.

### Differentiated mDPSC express L-type voltage gated Ca^2+^ channels

**Figure 5:**
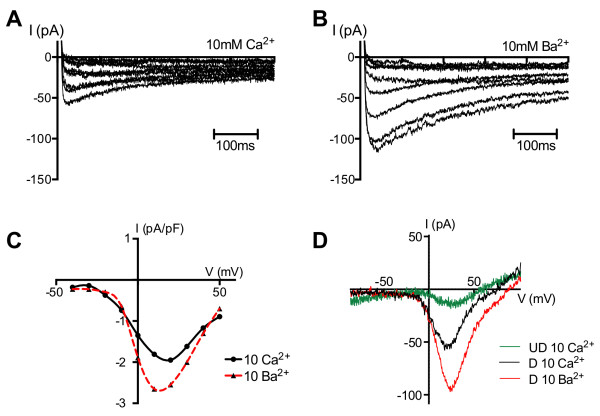
**Differentiated mDPSC express voltage-gated Ca**^**2+ **^**currents. A)** Representative whole cell patch clamp recording of an isolated differentiated mDPSC with multiprocessor neuronal morphology showing typical voltage-gated Ca^2+^ current in response to increasing voltage steps; 500 ms voltage steps were applied from -50 to +50 mV in 10 mV increments. **B)** There is a two-fold increase in current amplitude in response to the addition of 10 mM Ba^2+^. **C)** The current–voltage relationship of voltage-dependent current from one representative cell with neuronal morphology showing sensitivity to 10 mM Ba^2+^ addition. The amplitude of the current was normalised to cell capacitance. **D)** Representative L-type Ca^2+^ currents recorded from undifferentiated mDPSC (UD, green) and differentiated DPSC (D, black) in the presence of 10 mM Ca^2+^ and following the addition of Ba^2+^ (D, red), in response to 100 ms voltage ramps from -120 to +120 mV. mDPSC, murine dental pulp stem cells.

To determine the presence of neuron-specific ion channels, whole cell patch clamp analysis was performed on undifferentiated mDPSC and mDPSC following neural induction. We found voltage-gated L-type calcium channels in 21 of 27 cells with neuronal-like morphology. Figure 5 shows current traces from a representative neuronal-like differentiated cell recorded in response to 500 ms voltage steps in the presence of 10 mM Ca^2+^ (Figure 5A) or 10 mM Ba^2+^ (Figure 5B) in the bath solution. Figure 5C shows the current–voltage relationship of the Ca^2+^ current from the same cell. The current amplitude increased more than two-fold upon Ba^2+^ addition. To normalise the measured currents for variable cell size, the changes in amplitude were expressed as changes in current densities (pA/pF). In contrast, undifferentiated mDPSC produced only small L-type Ca^2+^ currents in response to 100 ms voltage ramps from -120 to +120 mV with an amplitude much lower than that from differentiated mDPSC (Figure 5D). No evidence of TTX-sensitive voltage-gated Na^+^ channels (n = 6) or TEA-sensitive K^+^ channels (n = 6) was observed in differentiated cells.

### Networks of neuronal-like differentiated mDPSC do not demonstrate action potentials

**Figure 6:**
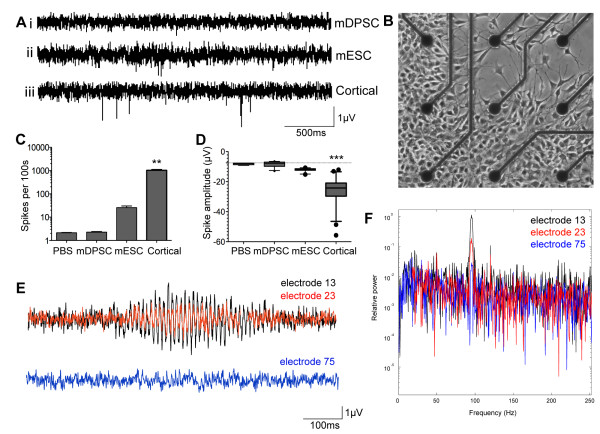
**Extracellular electrophysiology of differentiated mDPSC.** Twelve differentiated mDPSC cultures were assayed on MEAs and assessed for network activity. **Ai)** Differentiated mDPSC cultures exhibited numerous short, low amplitude events that surpassed a threshold of detection. These events were smaller and less numerous than those seen in mESC **(Aii)** and cortical **(Aiii)** cultures. **B)** A representative image of a central region of nine electrodes of a MEA with mDPSC at day 11 of differentiation. Electrode diameter is 30 μm. **C)** Maximum spike rate of mDPSC events within a 100 second bin was significantly lower than mESC and cortical signals and not distinguishable from PBS-only events. **D)** Boxplots of the mean amplitude of spike events. Spike amplitides were significantly lower in mDPSC cultures than control mESC or cortical cultures and were not significantly different from PBS-only control traces. **E)** A representative trace of a single oscillation event observed in a mDPSC MEA culture from day 32 of differentiation. The epicenter of oscillatory activity occurred at electrode 13 (e13, black) and was repeated at numerous adjacent electrodes, such as e23 (red), in phasic synchrony. A distant electrode, e75 (ii, blue), did not show any obvious oscillatory activity. **F)** The spectral power density of oscillatory activity in 6E reveals its broad frequency range peaking at 95 Hz that is consistent across the three representative electrodes shown. e13 (red) has the greatest intensity as observed visually followed by e23 and also e75 whose oscillations could not be detected by eye. **P <0.01, ***P <0.0001. ESC, muring embryonic stem cells; mDPSC, murine dental pulp stem cells; MEA, microelectrode array.

To investigate the network electrophysiology of differentiated mDPSC, cells underwent neuronal induction on MEAs (n = 12 cultures). Each MEA was assessed for extracellular electrical activity between days 10 and 20 with one culture surviving to 34 days. Measurements were taken a maximum of once every two days as increased use led to cell death and infection. Standard noise levels were recorded at 5 to 8 μV due to the small 30 μm electrodes of MEAs. Numerous spike events were identified in all 12 differentiated mDPSC cultures that satisfied the parameters of an action potential according to predetermined settings of amplitude, duration and shape. Figure 6Ai shows a representative singe electrode trace from one mDPSC-derived culture at differentiation day 16, such as that seen in Figure 6B. It shows one event that passed the spike detection threshold over the four second period. These events were common across many electrodes and had an average 1 ms duration, however they were insensitive to 10 μM TTX administration (n = 4 TTX controls, data not shown). Figure 6Aii and 6Aiii show representative traces of neuronally differentiated murine embryonic stem cells (mESC) and murine cortical cultures, respectively, each of which had larger and more frequent spike events. Average maximum spike rate per 100-second recording for mDPSC-derived cultures was 2.26 (± 0.751) spikes and was not significantly different from control PBS-only cultures (2.12 ± 0.335, *P* >0.05, Figure 6C). Cohen’s *d* demonstrated a small effect size between groups; *d* = 0.019 (-0.61,1.01). Comparatively, the maximum spike rates of differentiated mESC and cortical cultures were both higher at 25.75 ± 17.31 (*P* >0.05) and 1,028 ± 229 (*P* <0.001) spikes per 100 seconds, respectively. The effect size of each comparison was very large (mESC; *d* = 2.52 (1.64,3.51); cortical cells; *d* = 10.12). Furthermore, as can be seen in Figure 6A, event amplitude of mDPSC cultures was far smaller than that of the other cell types. Events from mDPSC cultures had an average amplitude of -8.38 ± 1.77 μV, which was not distinguishable from PBS-only cultures (-8.12 ± 0.59 μV, *P* >0.05, *d* = 0.16 (-1.0,0.68)). By contrast, the average amplitude of mESC events was greater at -12.08 ± 1.1 μV (*P* >0.05, *d* = 2.52 (1.78,3.33)) and cortical cultures significantly greater at -25.75 ± 8.57 μV (*P* <*0*.001, *d* = 2.45 (1.82,3.13)) per 100 second bin (Figure 6D). From these data we conclude that the events observed in mDPSC-derived neural cultures were not spontaneous action potentials.

We recorded oscillatory-like electrical activity over a population of local electrodes on day 32 (n = 1 culture). Each oscillation event lasted approximately 400 ms and occurred up to five times per 100 second recording. The epicenter of the oscillations occurred at a single electrode (e13), which had the greatest amplitude of activity (Figure 6E). Noise levels between oscillation periods on this electrode (e13) were similar to those of the surrounding electrodes. Numerous nearby electrodes supported the same oscillatory patterns but with reduced amplitude. Figure 6E shows representative traces of one oscillation event from e13, an adjacent electrode, e23, and a distant electrode over 1 mm away, e75, which does not display an obvious oscillatory pattern. The overlay of e13 (black) with adjacent e23 (red) showed that the electrical oscillations from both electrodes were in phase. The power spectral density demonstrated a broad frequency peak at 95Hz in both e13 and e23 (Figure 6F). Interestingly, the control trace from e75 also showed a smaller peak at the same frequency, indicative of weak electrical spread across the entire MEA.

## Discussion

We have shown that DPSC derived from mouse incisors give rise to immature neuronal-like cells. Following neuronal induction, mDPSC expressed neuronal cytoplasmic proteins, neurotransmitter-specific markers and functional voltage-gated L-type Ca^2+^ channels. The majority of mDPSC developed over time into networks with high gap junction protein expression and did not demonstrate spontaneous action potentials. These data suggest that mDPSC undergo neuronal differentiation *in vitro,* but to a limited maturity with properties more consistent with early neuronal development.

We identified that the majority of undifferentiated mDPSC expressed nestin and ßIII-tubulin which suggests a neurogenic potential; however, maintenance of the expression of these proteins in the differentiated cultures suggested continued immature phenotype as supported by electrophysiological data. This study, to the best of our knowledge, presents the first evidence of neuronal differentiation of DPSC from murine incisors. Following neural induction, mDPSC expressed the more mature neural markers NFM and GFAP. Consistent with other published findings, we also observed that mDPSC-derived neural cells co-expressed neuronal and glial markers [21]. It is not uncommon for these seemingly distinct markers to be co-expressed in the developing, but not the mature, CNS [22,23], thereby providing further evidence for the early stage of neural development of these cells. In summary, the immunophenotype of neural cells derived from mDPSC indicated a mixture of central and peripheral nervous system cell types. This was supported by expression of GFAP and S100, a marker of central and peripheral glial cells. In addition, we found that neuronal-like cells expressed cholinergic, GABAergic and glutaminergic markers. The differentiation of stem cells from human deciduous tooth dental pulp into dopaminergic neuron-like cells *in vitro* has previously been reported [24], indicating the potential for directing the differentiation of DPSC toward a central lineage.

From the neural inductive protocol used throughout these experiments we propose that mDPSC undergo neuronal differentiation *in vitro* but to a limited maturity. This is supported by high expression of a gap junction protein, Cx43, lack of synapsin expression, functional Na^+^ and K^+^ ion channels and a lack of spontaneous action potentials. We found clusters of cellular networks dominated by gap junctions rather than synapse protein expression. *In vivo* nervous system development proceeds from primitive gap junction signalling and later, during maturation, is superseded by synaptic signalling [25]. Cx43, in particular, is involved with neural precursor proliferation, neural differentiation and neurite outgrowth [26-29]. Moreover, Cx43 blockade decreases the rate of mature neuronal development in the mouse P19 carcinoma cell line, indicating its central role in nervous system maturation [27]. It is unclear what role is retained by connexins expressed in mDPSC-derived neural networks, as we saw no evidence of dye coupling, as would be expected if functional gap junctions were present. Non-channel functions of connexins are also of central importance to neural development. They are involved in cell-to-cell adhesion [30] and small molecule release through hemichannels, which is important for functions such as cell migration and neurite outgrowth [29,31]. In particular, hemichannel-mediated ATP release stimulates Ca^2+^ waves in early neural development, which has been linked with motoneuron, axon and dendritic development [32,33].

Differentiated mDPSC did not produce spontaneous action potentials or express the ion channels necessary to support them. Rather, we found an abundance of L-type voltage-gated Ca^2+^ channels in differentiated cells, in contrast to low levels of Ca^2+^ currents in undifferentiated mDPSC. Ca^2+^ channels are known to be abundant in developing cortical neurons [34,35], and calcium transients have been shown to be an important regulator of neurogenesis and neurite extension [36,37], often dependent on L-type calcium channel signaling [34]. In contrast, human DPSCs reliably developed voltage-gated Na^+^ and K^+^ currents *in vitro* using the same and another differentiation protocol [2,5], highlighting an important distinction between DPSC from different species. We made an isolated interesting observation that in a more mature mDPSC culture there were oscillatory electrical patterns in the gamma frequency range. This suggests the possible development of spontaneous network activity as found *in vivo*. It is well understood that oscillations are central to the development of neural networks during embryogenesis and early postnatal development; however, these are typically of a lower beta frequency range [25,38]. Gap junctions are intrinsic to sustaining such neural oscillations, as gap junction blockade causes a reduction or cessation of oscillatory activity [25,39]. Gap junction signalling may be responsible for the oscillations seen in this study but this was not empirically tested. We suggest that these oscillations may demonstrate the emergence of early electrical activity within networks of developing mDPSC. We made multiple attempts to pursue this *in vitro* observation but there were technical difficulties with maintaining these cultures long-term on MEA surfaces.

In contrast to that reported during human DPSC neuronal differentiation [5], we found that mDPSC numbers initially increased during the plating and epigenetic reprogramming stages of differentiation, likely due to continued progenitor cell proliferation. Following the removal of media serum and the addition of specific PKC agonists and neural growth factors, cell numbers declined. We suggest this may be due to the death of cells that did not have the intrinsic potential to respond to neuronal induction as well as the arrest of cell proliferation caused by long term PKC activation and the removal of serum [40]. Our observation of a stable cell number during the maturation stage of induction suggests that remaining cells showed neither a net loss nor gain during this stage of the protocol.

Alternative methods of neural induction may be considered for more efficient differentiation of mDPSC. Neurosphere generation, for example, may provide a microenvironment more reminiscent of *in vivo* development to support mature neural differentiation as seen previously with rat incisor and human DPSC [16,41].

## Conclusions

In conclusion, we have successfully generated neuronal-like cells from murine incisor DPSC to an immature stage of development. Our findings encourage the use of mDPSC to develop mouse models of autologous neural therapeutic transplantations for pre-clinical studies.

## Abbreviations

α-MEM: alpha-modified Eagle’s medium; ANOVA: analysis of variance; ChAT: choline acetyltransferase; CsGlutamine: caesium glutamine; Cx43: connexin 43; DAPI: 4’,6-diamidino-2-phenylindole; DPSC: dental pulp stem cell; (D)MEM: (Dulbecco’s) modified Eagle’s medium; EGTA: ethylene glycol tetraacetic acid; ER: epigenetic reprogramming; ESC: embryonic stem cell; FBS: fetal bovine serum; FGF: fibroblast growth factor; GAD65/67: glutamic acid decarboxylase 65/67; GFAP: glial fibrillary acidic protein; GTP: guanosine triphosphate; hDPSC: human dental pulp stem cell; hFF: human foreskin fibroblasts; IBMX: 3-isobutyl-1-methylxanthine; IHC: immunohistochemistry; MCS: multi channel systems; mDPSC: murine dental pulp stem cell; MEA: microelectrode array; mESC: murine embryonic stem cells; ND: neuronal differentiation; NFM: neurofilament-medium chain; NGF: nerve growth factor; NM: neuronal maturation; NT-3: neurotrophin 3; PBS: phosphate-buffered saline; PFA: paraformaldehyde; PKC: Protein Kinase C; TEA: Tetraethylammonium; TEM: transmission electron microscopy; TH: tyrosine hydroxylase; TPA: phorbol 12-myristate 13-acetate; TTX: tetrodotoxin; vGlut2: vesicular glutamate transporter 2.

## Competing interests

SAK currently receives funding from Mesoblast. All experiments were performed prior to the establishment of this partnership and Mesoblast had no input into experimental conceptualisation, interpretation of data or manuscript review. All other authors declare that they have no competing interests.

## Authors’ contributions

KME contributed to experimental design, performed all experiments, collected, analysed and interpreted data and wrote the manuscript. DCO was involved in experimental conceptualisation and design, supervision of work, data interpretation and manuscript review. MDL was involved in experimental conceptualisation and design, isolation of murine dental pulp stem cells, supervision of work and manuscript review. GYR assisted in experimental design, provided intracellular electrophysiology expertise and assisted in manuscript review. SAK contributed to experimental conceptualisation and design, data interpretation and considerable manuscript review. All authors read and approved the final manuscript.

## Supplementary Material

Additional file 1 **Supplementary methods.** Preparation of murine cortical cultures and murine embryonic stem cells. Click here for file
