# Supplementary material for: Neurogenic potential of dental pulp stem cells isolated from murine incisors
Source: Stem Cell Res Ther. 2014 Feb 27;5(1):30. doi: 10.1186/scrt419 (PMC4055132; doi:10.1186/scrt419)
Supplement: Additional file 1 — Supplementary methods. Preparation of murine cortical cultures and murine embryonic stem cells. [file scrt419-S1.doc]

## Additional File 1 - Supplementary Methods

*Murine cortical cultures*

C57 Black pups at postnatal day 1 were euthanized by decapitation and cortical explants digested with 10 µg/mL DNase (BD Bioscience) and 0.15% trypsin for 20 min. Resulting cell solution was sieved and centrifuged for enzyme removal. Cells were resuspended and plated in Neurobasal medium containing 1x B27 and N2 supplements, 1x GlutaMAX , 50 U/mL penicillin, 50 µg/mL streptomycin (Sigma) and 10 ng/mL FGF-2 (mouse)(ProSpec) at 15,000 cells/cm^2^ onto laminin (0.02 mg/mL) and poly-L-lysine (0.01%) coated glass coverslips or microelectrode array surfaces. Cultures underwent immunohistochemical or electrophysiological analysis as described.

### Neuronal differentiation of murine embryonic stem cells (mESC) on microelectrode arrays (MEAs)

mESC were underwent *in vitro* neuronal differentiation according to a protocol previously described (Ying and Smith, 2003). Briefy, mESC were maintained in ESC medium containing 10% foetal bovine serum, 2 mM L-Glutamine, 100 µM ß-mercaptoethanol, 1x Leukaemia inhibitory factor, 50 U/mL penicillin and 50 µg/mL streptomycin (Sigma) in Dulbecco’s modified eagle medium (DMEM). For neuronal differentiation, mESC were dissociated with trypsin and 3-5x10^4^ cells replated onto the electrode region of 0.1% gelatin-coated MEAs in N2B27 medium containing 0.5x N2 and B27 supplements, 50 mg/mL bovine serum albumin, 25 mg/mL insulin, 100 µM ß-mercaptoethanol, 1 mM L-Glutamate, 50 U/mL penicillin and 50 µg/mL streptomycin in 50% Neurobasal medium, 50% DMEM/F12. All reagents were sourced from Gibco unless otherwise specified.
